# Relative age effect, morphological status and physical performance of U-15 Croatian football players

**DOI:** 10.1371/journal.pone.0327878

**Published:** 2025-07-14

**Authors:** Ante Rađa, Jakov Marasović, Marko Erceg, Peter Krustrup, Morten B. Randers, Luca Paolo Ardigò

**Affiliations:** 1 Faculty of Kinesiology, University of Split, Split, Croatia; 2 Department of Sports Science and Clinical Biomechanics, University of Southern Denmark, Odense, Denmark; 3 Danish Institute for Advanced Study (DIAS), University of Southern Denmark, Odense, Denmark; 4 Sport and Health Sciences, University of Exeter, Exeter, United Kingdom; 5 Department of Teacher Education, NLA University College, Oslo, Norway; University of Montenegro, MONTENEGRO

## Abstract

The relative age effect (RAE) refers to the unequal quartal-to-quartal birth distribution in competitive sports, leading to more athletes being born earlier in the selection period. This effect results in relatively older players, who are often physically more developed, being overrepresented in youth academies and professional levels, as they receive more attention and opportunities due to their advanced biological maturation and superior physical performance compared to their younger peers. This study involved 177 U-15 football players from Dalmatia, Croatia, averaging 14.1 ± 0.9 yrs of age and 6.6 ± 1.9 yrs of training experience. Anthropometric characteristics, motor-functional abilities, and specific abilities with the ball were measured over two days, using standardised equipment and procedures, and the data were analysed with descriptive statistics, one-way analysis of variance, and post hoc least significant difference tests to identify differences across quartiles. The study found statistically significant differences in the distribution and characteristics of players born in different quartiles of the year, with 63.3% born in the first two quartiles. RAE influenced young football players’ birth distribution and physical characteristics, with a higher proportion born in the first quartile of the year (Q1) and showing superior morphological development and physical performance. Q1 players were significantly taller and heavier and performed better in explosiveness tests (medium effect sizes) compared to other quartiles. Furthermore, small to medium effect differences occurred in circumferences, 20-m, 30-m sprints and ball-kicking velocities in favour of Q1. As these abilities and characteristics are key to success in football, players born in the first quartile have an advantage in the selection process. This highlights the need for coaches to focus on potential talent rather than current performance despite the competitive pressure to win.

## Introduction

The relative age effect (RAE), being the unequal quartal-to-quartal birth distribution within a selection year, has been described in research spanning multiple decades [1–5] in high-level sports [6–10]. This leads to more participants born earlier in the selection year than later, especially in talent academies [6,9,11]. The Fédération Internationale de Football Association currently holds January 1^st^ to December 31^st^ of a calendar year as a competitive category, which main goal is to make the competition more balanced and to give everyone a similar chance for success.

During childhood and adolescence, players born earlier in the selection year may exhibit more advanced physical attributes and abilities, which can cause differences in players’ performance and play a role in the selection process. Successful initial performance may subsequently cause higher expectations from the coaches and higher self-esteem for the players, which could lead to more opportunities and better training in elite clubs. Hancock et al. [12] suggested a theoretical model explaining how RAE works, highlighting the intertwined influence of parents, coaches and players during the selection process. Their model suggests that RAE becomes more pronounced because of these social influences, contributing to the over-representation of players born early in the selection period. This may cause, over time, over-representativeness of players born earlier in the calendar year in the professional sport and youth academies. Research shows a significant bias in player recruitment, with 37.6% of players born in the first quartile and only 13.2% in the last quartile [8]. Unequal birth distribution in academies goes as far as the pro level, and the greatest number of professional players are born in the first three months of the year [2,10,13,14]. This may be due to the “drop off” of younger players during youth development stages, as the physical advantages of being older than those they compete against are equalled during adulthood. Football matches consist of various actions in defence and offence, such as dribbling, sprinting, jumping, kicking, sliding, etc., where the ability to be faster, stronger and more agile could be advantageous in the selection process [13,15–19]. Players born in the first quartile are often physically more dominant than those born in the fourth quartile [2,4,15,18]. As these players are chronologically older, they are also likely to be the first individuals within their age group to experience the positive physical changes associated with puberty. These boys are typically taller, heavier, faster, stronger, and more powerful than their later maturing peers [2,8,18]. Therefore, players born in the first months of the calendar year, as they present greater chronological age, are more likely to be in the more advanced stages of biological maturation until after puberty [20], and, whereas this developmental head-start is particularly evident in pre-puberty, it continues to influence into puberty and even post-puberty.

However, Matta et al. [16] reported no significant differences between birth quartiles in physical performance, including aerobic running, jump tests, sprint performance and technical performance. An interesting observation by Deprez and colleagues was an overrepresentation of earlier maturing boys in the later born quartile compared to earlier born quartile in which later maturing boys were more prevalent [8,19], which may, in some cases, have made up for the disadvantage of being born later in the selection year. The selection bias towards early-born players, as observed across many studies, may arise before puberty, as relatively older players are commonly more likely to be selected for talent identification and development programmes, as they receive more attention from the coaches from an early age, resulting in more playing minutes [5,21–24]. Thus, selection biases towards players born in the first quartile of the year have been well-established, especially in professional football academies, emphasising identifying and developing the most talented youth [22,25].

These findings underscore the need for further research into RAE and its impact on youth football players’ morphological characteristics and physical performance. A more nuanced understanding of these issues may benefit coaches and scientists, allowing for a fairer selection and talent identification process. Therefore, the main objectives of this study were to 1) determine the presence of RAE in U-15 youth Croatian football players and 2) evaluate the influence of possible RAE on morphological status and physical performance in these players.

## Materials and methods

### Study design

This was a cross-sectional study with two main objectives: 1) to determine the presence of RAE in youth football players and 2) to evaluate the influence of RAE on morphological status and physical performance. In order to comply with the study objectives, the study included a thorough 2-day testing protocol. The study included nine football clubs, with three clubs representing each of three different levels of competition in the U-15 category (1^st^ and 2^nd^ Croatian Football League South Division and 1^st^ County League). Informed written consent was obtained from the participants’ parents or guardians, who were thoroughly informed about the study’s purpose, benefits and potential risks. The consent forms were explicitly approved by the “The Ethical Committee of the Faculty of Kinesiology” (Split, Croatia). Shared data were in accordance with participant consent and all applicable local laws. The study adhered to the ethical standards of the 1964 Helsinki Declaration and its subsequent amendments. The inclusion criteria for study participation were: i) participation in at least 85% of the training sessions, ii) regularly participating in the previous competitive seasons, iii) having a valid sports medical certification and iv) being healthy (no pain or injury) and not consuming any drugs. All players had Croatian Football Federation identity cards signed and were fully healthy and medically examined by a local sports specialist doctor. To minimise potential interference with the experiment, participants refrained from consuming caffeine-containing beverages for 24 hours and abstained from eating for 2 hours before testing.

### Participants and setting

The sample of respondents for this research was 177 young U-15 football players from Dalmatia, Croatia, with an average age of 14.1 ± 0.9 yrs and 6.6 ± 1.9 yrs of training experience. Participant recruitment occurred in the second half of May during the 2015/2016 competition season (starting 16/05/2016 and ending 31/05/2016), and the current research took place in June at the end of the same competition season. Each participant completed all trials simultaneously on the testing days and under consistent climate conditions (8–10 a.m., 25.6 ± 0.8˚C temperature and 36.3 ± 2.5% relative humidity). Each test started with the participants filling out a questionnaire related to the date of birth, dominant leg and training experience. Due to the scope of the research, the testing was carried out over two days.

### Procedures

Variables used to assess the anthropological status of young football players were divided into three groups: anthropometric characteristics, motor-functional abilities and specific abilities with the ball (Table 1). The birth quartile of the players was independent variable while variables used to determine the morphological status and physical performance of the players were dependent variables.

**Table 1:** Variables used in research.

Anthropometric characteristics	Motor-functional tests	Specific tests with the ball
Knee diameter (KD)	Standing long jump (SLJ)	20 meters with the ball (20m B)
Elbow diameter (ED)	Sargent test (ST)	Slalom test with the ball (SLAL B)
Upper arm circumference (UAC)	Medicine ball throw (MBT)	Zig-zag test with the ball (ZIG-ZAG B)
Lower leg circumference (LLC)	Slalom test (SLAL)	93639 test with the ball (93639 B)
Upper arm skinfold (UAS)	Zig-zag test (ZIG-ZAG)	Instep kick dominant leg (IKD)
Back skinfold (BS)	93639 test turnaround (93639)	Instep kick non-dominant leg (IKN)
Suprailiac skinfold (SS)	5-meter sprint (5m)	
Lower leg skinfold (LLS)	10-meter sprint (10m)	
Body fat percentage	20-meter sprint (20m)	
Body mass	30-meter sprint (30m)	
Body height	Yo-Yo Intermittent Recovery	
Sitting height	test level 1 (YYIR1)	
Body mass index (BMI)		

### Anthropometric characteristics

On the first day, variables to assess anthropometric characteristics (height, sitting height, weight, diameters, circumferences and skinfolds) were measured (listed in Table 1). Anthropometric data were measured with a portable stadiometer (SECA, Leicester, UK; for height) and an electronic scale (HD-351, Tanita, Arlington Heights, USA; for body mass). The circumferences of the upper arm (UAC) and lower leg (LLC) were measured using a tape. Two diameters (in cms) were obtained using a Vitruvian caliper (Harpenden, England): elbow diameter (ED) and knee diameter (KD). Triceps, subscapular, abdominal and lower leg skinfold thickness (mm, to the nearest 0.1 mm) were measured, using a skinfold caliper (Harpenden, England). The Body Mass Index (BMI) was calculated from height and body weight (kg/m^2^), while Faulkner’s formula [26] was used to estimate the fat percentage: Fat percentage = the sum of four skinfolds (tricipital + subscapular + suprailiac + abdominal) • 0.153 + 5.783. Pubertal timing was estimated according to each individual’s biological age of maturity as described by Mirwald and colleagues [27].

### Motor tests with and without the ball

After anthropometric measurements, the following tests were conducted: A 30-m sprint test with 5-, 10- and 20-m split times, 20-m sprint with ball, along with three tests to assess agility: slalom test (SLAL) with and without ball, zig-zag test (ZIG-ZAG) with and without ball, and 93639 test with 180° change of direction with and without ball. Slalom test consisted of six cones at a distance of 2 m between them were placed in an 11 m straight line [28]. The participant’s task was to make a slalom between the cones at maximum speed forward and backward with a turn around the last cone. ZIG-ZAG test course consists of four cones placed on the corners of a rectangle 10 by 16 feet, with one more cone placed in the center. If the cones are labeled 1–4 around the rectangle going along the longer side first, and the center cone is C, the test begins at 1, then around C, 2, 3, C, 4, then back to 1. Test 93639 is a test with 180° change of direction. Participants were required to run 9 m from the starting line A to line C. After touching line C with one foot, a 180° turn was made and they ran 3 m to line B. The participants then ran 6 m to line D, made another 180° turn and ran 3 m to line C. Lastly, they made a final 180° turn and ran 9 m to line E.

The timed tests were recorded using a photocell system (Brower Timing Systems, Draper, United States). Participants were placed 50 cm behind starting line for each test in order to eliminate possible interference with photocells system during start. Each participant had a 3-minute recovery period after each attempt.

### Explosive power

On the second day of testing, participants performed tests to assess basic and specific explosive power with a ball: 3-kg medicine ball throw (MBT), standing long jump (SLJ), Sargent test (ST), instep kick with dominant (IKD) and non-dominant leg (IKN). MBT was performed after participants stood behind a line with slightly widened feet in the squat position with a 3 kg rubber medicine ball. Participant’s task was to perform a maximal take-off to stretch the whole body to throw the medicine ball as far as possible from the chest. For SLJ participants stood behind the line and had to jump as far as possible horizontally. For ST participants were asked to stand sideways on the wall and raise the dominant hand’s marked fingertips to a maximum height on the wall without lifting the heels. The participant’s task was to perform a vertical jump on two legs, touching the wall at the highest possible point. The difference between the two points was the test result. IKD and IKN were performed from the 11-m spot. The participants were instructed to shoot the ball as fast as they could and straight to the center of the goal. A radar (Pocket Radar, Inc., Santa Rosa, California; with ±2 km·h^-1^ accuracy) was used to determine ball velocity.

### Aerobic endurance

The final test of the second day was the Yo-Yo Intermittent Recovery test level 1, used to assess aerobic capacity as described by Krustrup et al. [29].

### Statistical analysis

Basic descriptive statistics including means, standard deviation (SD) and range (minimum and maximum) results were calculated. The normality of the variables was tested using the Shapiro–Wilk test, and results confirmed that the assumption of normality was not violated. The chi-squared ($\chi^{2}$ ) test was used to determine differences between the expected frequency of players born and the observed frequency of players born in a certain quartile. After confirming the homogeneity of variances with Levene’s test, a one-way analysis of variance (ANOVA) was used to determine the differences between groups (quartiles) in all three variable categories: anthropometric characteristics, motor-functional abilities and specific abilities with the ball. Post hoc analysis (least significant difference test – LSD) was used for pairwise comparisons. Additionally, partial eta squared ($\eta^{2}$) was computed to estimate effect size in the ANOVA, with the following thresholds used for interpretation: small (≥ 0.01), medium (≥ 0.06), and large (≥ 0.14) [30]. The significance level was set at p < 0.05.

## Results

In Table 2, statistically significant differences ($\chi^{2}$ = 836.68 and p < 0.01) were found in birth quarter showing more players born in the first (Q1) than in the third (Q3) and fourth (Q4) quartiles (35.0/28.3/17.5/19.2% for Q1/second quartile [Q2]/Q3/Q4, respectively), which equals 63.3% of players born in the first half of the year compared to 36.7% in the second half. As expected, chronologically, the oldest players were those born in the first quartile and were significantly different (F_(3,173)_=14.86, p < 0.01 and $\eta^{2}$ = 0.20) from players born in all other quartiles. Age at peak height velocity (APHV) was not significantly different between the four birth quartiles (F_(3,173)_=2.45, p > 0.05 and $\eta^{2}$ = 0.04). Players born in the first quartile of the year had significantly more training experience (F_(3,173)_=3.12, p < 0.05 and $\eta^{2}$ = 0.05) compared to players born in the other quartiles (7.16 ± 1.79 vs 6.46 ± 1.88, 6.13 ± 1.89, 6.26 ± 1.75 yrs for Q2, Q3 and Q4, respectively).

**Table 2:** Distribution of birth date quartiles, differences between given groups in chronological age, age at peak height velocity and training age.

	Q1 n (%)	Q2 n (%)	Q3 n (%)	Q4 n (%)	Chi square test
Number of players	62 (35.0%)	50 (28.3%)	31 (17.5%)	34 (19.2%)	$\frac{\chi^{2}=836.68**}{\eta^{2}}$
Chronological age (yrs)**	14.4 ± 0.7 (¤&#)	14.1 ± 0.8 (#)	14.0 ± 0.8 (#)	13.4 ± 0.8	0.20
APHV	13.64 ± 0.60	13.89 ± 0.65	13.92 ± 0.47	13.71 ± 0.60	0.04
Years of training*	7.16 ± 1.79 (¤&#)	6.46 ± 1.88	6.13 ± 1.89	6.26 ± 1.75	0.05

Q1: first quartile, Q2: second quartile, Q3: third quartile, Q4: fourth quartile, ANOVA: analysis of variance, $\eta^{2}$: Eta-squared, APHV: age at peak height velocity. * and ** denote significant main effects at p < 0.05 and p < 0.01, respectively. ¤ denotes significantly different from 2^nd^ quartile, & denotes significantly different from 3^rd^ quartile and # denotes significantly different from 4^th^ quartile (p < 0.05).

Table 3 shows the differences in morphological variables between birth quartiles. In short, significant difference was observed in body height (F_(3,173)_=6.74, p < 0.01 and $\eta^{2}$ = 0.10)), sitting height (F_(3,173)_=7.26, p < 0.01 and $\eta^{2}$ = 0.11), body mass (F_(3,173)_=7.15, p < 0.01 and $\eta^{2}$ = 0.11) and BMI (F_(3,173)_ =3.76, p < 0.05 an $\eta^{2}$ = 0.06) with post hoc test revealing players from Q1 being taller, having higher sitting height, body mass and body mass index than players from Q2, Q3 and Q4 with no difference between other birth quarters (Table 3). In addition, upper arm circumference was significantly different (F_(3,173)_=4.94, p < 0.01 and $\eta^{2}$ = 0.08) with Q1 being bigger than all other birth quarters. A significant difference was observed for lower leg circumference (F_(3,173)_=3.17, p < 0.05 and $\eta^{2}$ = 0.05) with a larger circumference for Q1 than Q3 and Q4 (Table 3).

**Table 3:** Morphological characteristics of U-15 football players according to birth quartiles.

	Q1 (n = 62)	Q2 (n = 50)	Q3 (n = 31)	Q4 (n = 34)	$\eta^{2}$
Body height (cm)**	172.5 ± 8.8 (¤&#)	167.5 ± 10.4	166.9 ± 9.4	163.8 ± 9.8	0.10
Sitting height (cm)**	88.9 ± 5.4 (¤&#)	85.5 ± 5.7	85.0 ± 4.6	84.5 ± 5.0	0.11
Body mass (kg)**	59.8 ± 10.2 (¤&#)	54.1 ± 10.3	52.7 ± 9.3	51.0 ± 9.7	0.11
Body mass index (kg/m²)*	19.9 ± 2.0 (¤&#)	19.1 ± 1.9	18.8 ± 1.9	18.8 ± 2.1	0.06
KD (cm)	9.6 ± 0.5	9.4 ± 0.5	9.4 ± 0.6	9.4 ± 0.5	0.02
ED (cm)	6.3 ± 0.4	6.1 ± 0.5	6.1 ± 0.4	6.2 ± 0.5	0.02
UAC (cm)**	27.2 ± 2.7 (¤&#)	26.1 ± 2.3	25.5 ± 2.5	25.4 ± 2.6	0.08
LLC (cm)*	35.1 ± 2.6 (&#)	34.0 ± 2.8	33.6 ± 2.8	33.5 ± 3.0	0.05
UAS (mm)	8.0 ± 2.1	8.5 ± 2.2	8.7 ± 2.9	9.4 ± 3.1	0.03
BS (mm)	7.9 ± 1.7	7.8 ± 1.9	7.6 ± 1.9	7.7 ± 2.5	0.00
SS (mm)	7.4 ± 2.7	7.2 ± 2.6	7.4 ± 2.7	7.7 ± 3.6	0.00
LLS (mm)	8.9 ± 2.1	9.3 ± 2.6	9.6 ± 2.9	9.8 ± 2.4	0.02
Body fat (%)	13.4 ± 2.8	14.1 ± 3.1	14.5 ± 3.9	15.1 ± 4.1	0.03

KD: knee diameter, ED: elbow diameter, UAC: upper arm circumference, LLC: lower leg circumference, UAS: upper arm skinfold, BS: back skinfold, SS: suprailiac skinfold, LLS: lower leg skinfold. * and ** denote significant main effects (p < 0.05 and p < 0.01, respectively). ¤-significant differences between 1^st^ and 2^nd^ quartile, &-significant differences between 1^st^ and 3^rd^ quartile and #-significant differences between 1^st^ and 4^th^ quartile.

Table 4 shows the performance in motor-functional abilities for players born in different birth quartiles. A significant main effect was observed for SLJ, ST, MBT, 20m, 30m and 93639m, with no differences in the other tests. In SLJ and MBT, players born in Q1 performed significantly better than those born in Q2, Q3 and Q4, whereas in 30m and 93639m, players in Q1 performed better than in Q2 and Q4. In ST, Q1 players performed better than Q3 and Q4, whereas in 20m Q1 performed better than Q4, with no other differences between groups observed for the seven variables.

**Table 4:** Physical performance of U-15 football players according to birth quartiles.

	Q1 (N = 62)	Q2 (N = 50)	Q3 (N = 31)	Q4 (N = 34)	$\eta^{2}$
SLJ (m)**	2.08 ± 0.18 (¤&#)	1.98 ± 0.20	1.99 ± 0.16	1.94 ± 0.16	0.08
ST (cm)**	42.8 ± 6.2 (&#)	40.6 ± 6.4 (#)	39.8 ± 5.8	37.8 ± 5.6	0.08
MBT (m)**	8.75 ± 1.59 (¤&#)	7.75 ± 1.67	7.66 ± 1.53	7.48 ± 1.23	0.11
5m (s)	1.07 ± 0.07	1.08 ± 0.07	1.09 ± 0.06	1.10 ± 0.07	0.03
10m (s)	1.86 ± 0.09	1.89 ± 0.10	1.87 ± 0.07	1.90 ± 0.09	0.03
20m (s)*	3.27 ± 0.18 (#)	3.34 ± 0.21	3.35 ± 0.17	3.38 ± 0.19	0.05
30m (s)*	4.62 ± 0.24 **(¤#)**	4.72 ± 0.28	4.71 ± 0.26	4.77 ± 0.24	0.05
SLAL (s)	7.45 ± 0.31	7.60 ± 0.45 (#)	7.48 ± 0.37	7.41 ± 0.53	0.03
ZIG-ZAG (s)	6.46 ± 0.37	6.58 ± 0.35	6.48 ± 0.34	6.57 ± 0.44	0.02
93639m (s)*	7.69 ± 0.38 (¤#)	7.92 ± 0.49	7.81 ± 0.36	7.89 ± 0.40	0.05
Yo-Yo IR1 (m)	1243 ± 469	1114 ± 428	1141 ± 388	1034 ± 454	0.03
Estimated VO_2_max (ml·min^-1^·kg^-1^)	46.9 ± 3.9 **(#)**	45.8 ± 3.6	46.0 ± 3.3	45.1 ± 3.8	

SLJ: standing long jump, ST: Sargent test, MBT: medicine ball throw, SLAL: slalom test, ZIG-ZAG: zig zag test, 93639m: 93639 test with turnaround, VO_2_max: maximal oxygen uptake. * and ** denote significant main effects (p < 0.05 and p < 0.01, respectively). ¤ denotes significantly different from 2^nd^ quartile, & denotes significantly different from 3^rd^ quartile and # denotes significantly different from 4^th^ quartile (p < 0.05).

Table 5 presents specific football technical abilities. Significant differences were observed for IKD and IKN (F_(3,173)_=5.47, p < 0.01, $\eta^{2}$ = 0.09 and F_(3,173)_=7.114, p < 0.01, $\eta^{2}$ = 0.11, respectively) as well as 93639m B (F_(3,173)_=2.94, p < 0.05, $\eta^{2}$ = 0.05). For IKD and IKN, Q1 players performed significantly better than Q2, Q3 and Q4 players, whereas in 93639m B Q1 players performed better than Q2 and Q4 players.

**Table 5:** Football-specific abilities of U-15 football players according to birth quartiles.

	Q1 (N = 62)	Q2 (N = 50)	Q3 (N = 31)	Q4 (N = 34)	$\eta^{2}$
20m B (s)	3.44 ± 0.18	3.52 ± 0.24	3.50 ± 0.20	3.51 ± 0.20	0.02
SLAL B (s)	10.61 ± 0.76	10.72 ± 0.89	10.80 ± 0.61	10.63 ± 1.06	0.00
ZIG-ZAG B (s)	8.44 ± 0.45	8.53 ± 0.63	8.51 ± 0.42	8.43 ± 0.55	0.00
93639m B (s) *	9.65 ± 0.63 (¤#)	9.95 ± 0.65	9.82 ± 0.45	9.96 ± 0.65	0.05
IKD (km·h^-1^) **	95.1 ± 8.8 (¤&#)	89.6 ± 10.1	90.2 ± 8.4	88.2 ± 9.2	0.09
IKN (km·h^-1^) **	83.9 ± 9.5 (¤&#)	76.9 ± 9.7	76.6 ± 9.4	76.2 ± 11.6	0.11

20m B: 20 meters with ball, SLAL B: slalom test with ball, ZIG-ZAG B: zig-zag test with ball, IKD: instep kick with dominant leg, IKN: instep kick with non-dominant leg. * and ** denote significant main effects (p < 0.05 and p < 0.01, respectively). ¤ denotes significantly different from 2^nd^ quartile, & denotes significantly different from 3^rd^ quartile and # denotes significantly different from 4^th^ quartile (p < 0.05).

All individual data are available in the supplementary S1 File_file.xlsx.

## Discussion

This study provides strong evidence for the relative age effect (RAE) among U-15 Croatian football players, revealing a significant overrepresentation of players born in the year’s first quartile (Q1). Moreover, differences in morphological characteristics and physical performance were apparent and showed that earlier-born players had superior height, weight, and muscle mass, as well as greater performance in physical tests compared to their later-born peers. Only minor differences were observed in ball handling tests. These findings align with a growing body of literature highlighting the influence of RAE across various youth sports, particularly in football, where chronological age influences the developmental advantages that benefit players born early in the year [6,11,19]. In this study, 35% of players were born in Q1, with 19% born in Q4 and nearly two-thirds born in the first half of the year. These findings are in accordance with previous studies on RAE showing overrepresentation of players born in the first half of the year especially in Q1 [8,16,31]. These data underline the finding that coaches favor older players who are more developed than their later-born peers, even though the biological advantages for the early-born equal out after the growth spurt and puberty and into adulthood [4,5].

The study revealed that players born earlier in the year (Q1) exhibited more advanced morphological characteristics than those born later. Players in Q1 were, on average, 5–9 cm taller and 11–17% heavier than players in Q2, Q3 and Q4, with the biggest difference between Q1 and Q4 players. In addition, BMI and sitting height were higher in players from Q1 than in the other birth quarters. These observations align with Altimari et al. [32], who found significant differences in body mass and height in favor of older players when divided into tertiles. Similarly, Deprez et al. [19] found differences in body height between quartiles for the elite U15 players and Leyhr et al. [33] obtained a significant correlation between date of birth and body mass and body height. On the other hand, some studies [9,16] did not find differences between birth quartiles in body mass and height. In addition to height and body mass, significant differences were found in the upper arm and lower leg circumferences, with Q1 players having 4–7% larger upper arm circumferences than their Q2, Q3 and Q4 counterparts and 5% larger lower leg circumferences than Q3 and Q4. This reflects greater muscle mass, which may help explain the superior physical performance of Q1 players in this study. Moreover, in sports like football, where size and strength are critical for performance [34,35], these physical advantages of being older and more mature lead to selection bias from the coaches [6,22], which may partly explain the overrepresentation of early-born players.

Players born in Q1 outperformed their Q3 and Q4 peers in several physical performance tests, particularly those assessing explosive power and speed. Q1 players exhibited 5–7% greater jumping distance in the standing long jump (SLJ) than Q2, Q3 and Q4 players. Q1 players also threw the medicine ball 13–17% farther in the medicine ball throw than players from other birth quarters. In sprinting tests, Q1 players demonstrated 2–3% faster times over 30 meters than Q2 and Q4 players. Given that speed and strength are critical determinants of football performance, the advantages seen in Q1 players may explain why these players are more frequently selected for elite youth teams. These performance disparities are likely due to the combined effects of greater physical size and muscle mass. No significant differences in age at peak height velocity (APHV) across the birth quartiles were found. This suggests that the superior physical performance of Q1 players may primarily be due to the higher chronological age, as the Q1 players are further into their biological maturation.

In addition to physical performance, better performance was observed in Q1 players for some football-specific skill tests. Q1 players performed better in kicking tasks, with their dominant and non-dominant legs showing 5–8 and 9–10% greater velocity, respectively, than their Q4 counterparts. Kicking ability is a key technical skill in football, and the superior performance of Q1 players may be partly explained by their greater muscle mass and strength, which enhance their capacity for power-based tasks like kicking. We found no difference between birth quartiles in 3 out of 4 ball-handling skills such as the slalom and zig-zag dribbling tests. It is possible that ball-handling skills are more influenced by training and experience than by the physical advantages associated with being born earlier in the year [4,16]. However, in our study, Q1 players also had more years of training than their younger peers, which was insufficient to differentiate between birth quartiles’ ball-handling test performances.

The overrepresentation of Q1 players and their superior physical performance suggest that RAE may lead to the unintentional exclusion of talented later-born players as coaches may focus too much on the current physical performance level highly, which is highly influenced by the maturity and chronological age advantages for Q1 players [19,24,31,36]. This phenomenon is particularly concerning, as RAE has been shown to persist into adulthood in many sports, limiting opportunities for later-born players to reach elite levels [10,36]. Coaches, scouts and football academies should be aware of these potential biases and take steps to address RAE during the talent identification and development process. One possible solution is to implement age-grouping strategies based on biological maturation rather than chronological age [19]. Additionally, coaches could adopt a more holistic approach to talent identification [8,10], focusing on long-term development rather than short-term success. Focusing solely on the current season and/ or upcoming match may bring success or a win in the short term. However, a more elaborate approach during talent selection and especially during talent identification could produce huge effects in the long run. Some authors [6,8,10] proposed that coaches should prioritise technical skills, tactical understanding and psychological resilience, which may help mitigate the effects of RAE and ensure that all players, regardless of their birthdate, are allowed to succeed.

When assessing RAE during selection process within youth academies, valuable information could be obtained. That way, potential loss of talents could be somewhat mitigated (underestimation of quality for players born later). Also, it enhances coaches and scouts with more information about every individual player. In a way, when taking RAE into account it can even prevent an overestimation of some players (i.e., born earlier in the calendar year).

The study has some limitations. E.g. we did not take other influencing variable such as training intensity, specific focus of the various clubs etc. into account, which may influence player development. It is possible that players in their designated clubs had different training intensities and focus for the training, which could have led to differences in certain characteristics or abilities. Moreover, studies have shown that certain physical attributes are related to specific positions with goalkeepers, defenders and attackers being the tallest and heaviest [37], which may also influence the development of certain ball handling skills. We did not take positions into account in our analysis, which may be a limitation. Also, even though participants had at least 3 minutes of recovery between attempts, we did not check for fatigue evidence (i.e., monitoring heart rate) during tests to assess agility with and without the ball (SLAL, ZIG-ZAG, 93639).

## Conclusions

In conclusion, this study confirms the presence of a relative age effect among U-15 Croatian football players, with players born earlier in the year demonstrating superior morphological characteristics and physical performance but not in 3 out of 4 ball-handling skills compared to those born later. The overrepresentation of Q1 players suggests that RAE may bias the selection process in youth football, potentially excluding talented later-born players. Players who demonstrate physical dominance in the game are more likely to be selected to play. Football academies and coaches should consider strategies to account for relative age differences in their talent identification processes to ensure a fairer and fairer development pathway for all players.

Coaches should pay more attention to identifying “potential” talent rather than focusing solely on “current” talent. Addressing RAE within clubs should generate more discussion regarding players’ playing potential from multiple perspectives. Ideally, it could lower the overestimation or the underestimation of current quality for players born earlier or later in the calendar year. Unfortunately, this may be one of those: “easier to say than done” as the majority of coaches and clubs are eager to win and dominate in the competition they play in.

## Supporting information

S1 File Raw Data. All individual data.
(XLSX)
